# Prevalence and associated factors of screen-positive scoliosis among primary and secondary school students in Taizhou, China: a cross-sectional analysis based on ATR thresholds with identification of the apparent 5° measurement clustering

**DOI:** 10.3389/fped.2026.1901184

**Published:** 2026-10-27

**Authors:** Han-tao Jiang, Guang Wang, Si-yu Yang, Feng-ze Wu, Ting-ting Tang, Wei-qiang Wang, Zhi-peng Wang, Ling-hong Liang, Xiao Huang, Yan Ge, Min-jiao Wu

**Affiliations:** 1 Department of Orthopedics, Taizhou Hospital of Zhejiang Province Affiliated to Wenzhou Medical University, Taizhou, Zhejiang, China; 2 Department of Rehabilitation, Taizhou Hospital of Zhejiang Province Affiliated to Wenzhou Medical University, Taizhou, Zhejiang, China; 3 Department of Social Rehabilitation Services, Taizhou Hospital of Zhejiang Province Affiliated to Wenzhou Medical University, Taizhou, Zhejiang, China

**Keywords:** adolescent idiopathic scoliosis, angle of trunk rotation, associated factors, measurement clustering, prevalence, school screening, scoliometer

## Abstract

**Background:**

Adolescent idiopathic scoliosis (AIS) affects 0.47%–5.2% of school-aged children globally. The angle of trunk rotation (ATR) measurement using a scoliometer is the primary screening tool, though threshold controversy exists between ≥5° and ≥7°. This study aimed to determine the prevalence of screen-positive scoliosis and its associated factors among students in Taizhou, China, with particular focus on exploring potential measurement clustering patterns at the 5° ATR threshold.

**Methods:**

A cross-sectional study was conducted among 813 students (ages 10–17 years) from four schools in Taizhou. Screening included general examination, Adam's forward bend test, ATR measurement with scoliometer, spinal movement test, and classification. The primary outcome was post-exercise ATR≥7°. Secondary outcomes included initial screening ATR≥5°. Univariate and multivariable logistic regression analyses were performed. Model performance was evaluated using AUC, Hosmer-Lemeshow test, and decision curve analysis.

**Results:**

Among 813 participants, 215 (26.4%) were classified as screen-positive: Grade I (5°≤ATR<7°): 165 (76.7%), Grade II (7°≤ATR<10°): 40 (18.6%), Grade III (ATR≥10°): 10 (4.7%). Significant clustering was observed at ATR = 5° (144/813 = 17.7%, ratio 5.5:1 compared to ATR 1–4°; *χ*^2^ = 794.88, *P* < 0.001 for deviation from interpolated expectation). The primary model showed female sex (OR = 3.509, 95%CI: 1.917–6.425, *P* < 0.001) and screen time (OR = 1.391, 95%CI: 1.070–1.809, *P* = 0.013) as significantly associated factors. BMI showed marginal significance (OR = 0.930, *P* = 0.052). A significant sex × screen time interaction was identified (OR = 1.68, *P* = 0.046). The main model demonstrated acceptable discrimination (AUC = 0.714) and calibration (HL *P* = 0.680).

**Conclusion:**

This study identifies statistically significant measurement clustering at the 5° ATR threshold (*χ*^2^ = 794.88, *P* < 0.001), which should be considered when interpreting screening results. Female sex and screen time were independently associated with screen-positive status, with screen time showing a stronger effect in females. Using ATR≥7° rather than ≥5° yields a substantially lower screen-positive rate (6.1% vs. 26.8%) that is more consistent with prevalence ranges reported in the literature, and may reduce unnecessary referrals and parental anxiety. However, these findings require validation against radiographic confirmation in future studies.

## Introduction

Adolescent idiopathic scoliosis (AIS) is a three-dimensional spinal deformity characterized by a coronal Cobb angle ≥10° on standing radiographs, often accompanied by vertebral rotation (1). AIS affects approximately 0.47%–5.2% of school-aged children worldwide, with significant geographic and demographic variations (2). The condition predominantly affects females, with a female-to-male ratio ranging from 1.5:1 to 5:1, particularly in curves exceeding 20° (3). In China, recent large-scale analyses indicate a scoliosis prevalence of approximately 1.2% among adolescents aged 10–18 years, with increasing trends over the past decade (4, 5).

Early detection through school-based screening programs is widely advocated, as timely intervention can prevent curve progression and reduce the need for surgical treatment (6, 7). The Adam forward bend test combined with scoliometer measurement of the angle of trunk rotation (ATR) is the most commonly used screening method owing to its simplicity, non-invasiveness, and acceptable reliability (8–10); however, its accuracy as a standalone diagnostic criterion has been questioned (11). In 2021, China incorporated scoliosis screening into routine student health examinations and published national technical guidelines for prevention and control of spinal curvature abnormalities (12).

However, controversy exists regarding the optimal ATR threshold for referral. International guidelines recommend various cutoffs: ≥5° (American Orthopaedic Society), ≥7° (International Society on Scoliosis Orthopaedic and Rehabilitation Treatment), or ≥10° (some European programs) (10, 13). This variability significantly impacts reported prevalence rates and referral patterns. In our own screening programs, ATR values appeared to cluster at exactly 5°, an observation that prompted the present analysis; to our knowledge, this apparent clustering has not been reported in the scoliosis screening literature.

Despite the widespread use of ATR-based screening, several gaps remain in the current evidence base. First, there is a lack of screening data from certain regions of China, including Taizhou in Zhejiang Province, where the prevalence and associated factors of screen-positive scoliosis have not been systematically characterized. Second, the impact of different ATR thresholds on prevalence estimates has not been directly compared within the same population, making it difficult to evaluate the trade-offs between sensitivity and specificity in the Chinese context. Third, although our preliminary screening experience suggested a possible clustering of ATR measurements at the 5° threshold, no published study has reported or quantified digit preference or threshold-related clustering in ATR measurement, so the phenomenon has not been systematically validated. Terminal digit preference—where measurements cluster at round numbers—is a well-documented phenomenon in clinical measurement (14, 15). These reports document round-number clustering in other clinical measurement contexts, such as vital signs and tumor diameter, and provide a plausible mechanistic analogy; they do not, however, constitute direct evidence of such clustering in ATR measurement, where data are commonly recorded in whole degrees and where 5° coincides with a widely used clinical cutoff. Addressing these gaps could inform the optimization of school-based screening protocols in China.

Several factors have been associated with AIS development. Female sex is the most consistently reported associated factor (2). Lower body mass index (BMI) has been identified in multiple studies (16–18), though this association may partly reflect detection bias, as BMI affects scoliometer readings, with higher BMI yielding lower ATR measurements (19). Increasing screen time and sedentary behavior have emerged as potential modifiable associated factors (20, 21), though evidence remains limited.

This study aimed to: (1) determine the prevalence of screen-positive scoliosis among primary and secondary school students in Taizhou, China; (2) explore potential measurement clustering patterns at the 5° ATR threshold and evaluate their impact on prevalence estimates; (3) evaluate associated factors using multivariable logistic regression; (4) assess the effectiveness of the spinal movement test in reducing false positives; and (5) provide evidence-based recommendations for ATR threshold selection in school screening programs.

## Materials and methods

### Study design and setting

This cross-sectional study was conducted from May to June 2026 in Taizhou, Zhejiang Province, China. The study protocol was approved by the Ethics Committee of Taizhou Hospital of Zhejiang Province Affiliated to Wenzhou Medical University (K2024121). Written informed consent was obtained from all participating students and their legal guardians prior to the screening.

### Participants

Students were recruited from two primary schools and two secondary schools in Taizhou. The inclusion criteria were: (1) students in grades 4 through 9 from the participating schools; (2) aged 10–17 years; and (3) provision of written informed consent by both the student and their legal guardian. The exclusion criteria were: (1) history of spinal surgery; (2) congenital spinal anomalies; (3) known neuromuscular diseases; and (4) inability to complete the screening procedure. A total of 813 students (408 males, 405 females) met the inclusion criteria and were enrolled in the study.

### Screening procedure

The screening procedure followed a standardized five-step protocol with established quality control measures. All students were examined in a private screening room with the back fully exposed. The primary examiner was a rehabilitation physician with 6 years of clinical experience, who performed the Adam forward bend test and scoliometer measurement. A rehabilitation attending physician with 8 years of clinical experience served as the supervising examiner, responsible for quality oversight, independent record verification, and re-measurement when necessary. Both examiners completed a standardized 1-hour training program prior to the study, conducted by a chief physician specializing in spinal surgery, which covered standardized student positioning during the Adam forward bend test, scoliometer placement at the point of maximum deformity, and ATR reading technique, followed by supervised practice sessions on volunteer students.

The five-step protocol comprised: (1) general visual inspection for shoulder asymmetry, scapular prominence, and pelvic tilt; (2) Adam's forward bend test to identify rib hump or lumbar prominence; (3) ATR measurement using a scoliometer (Scoliometer, Orthopaedic Systems Inc.) at the point of maximum deformity; (4) spinal movement test consisting of hanging from a horizontal bar for 30 s, followed by re-measurement of ATR; and (5) classification was based on the 2021 Chinese national guidelines. For each participant, the rehabilitation physician performed the primary ATR measurement. If the measurement was clinically questionable (e.g., inconsistent with visual inspection or clearly affected by patient movement), the attending physician performed a re-measurement, and the re-measured value was recorded as the final result. Students with post-exercise ATR ≥5° were advised to seek further medical evaluation, including standing posteroanterior radiographs for definitive assessment. This referral criterion was based on the 2021 Chinese national guidelines for adolescent spinal health screening. Hanging has been examined as a scoliosis-specific corrective exercise with measurable immediate effects on ATR (22).

Classification criteria were: Normal: no abnormalities on general examination, negative Adam's test, and ATR <5°; Poor posture: abnormalities on general examination OR positive Adam's test OR ATR≥5°, but maximum ATR <5° after the spinal movement test; Screen-Positive Grade I: 5°≤ post-exercise ATR <7°; Screen-Positive Grade II: 7°≤ post-exercise ATR <10°; Screen-Positive Grade III: post-exercise ATR ≥10°. The spinal movement test was performed only for students with initial ATR≥5°.

### Questionnaire

All participants completed a structured questionnaire covering demographic characteristics (age, sex, school type), anthropometric measurements (height, weight, BMI), growth and development (menarche age for females), family history of scoliosis, and lifestyle factors including bag carrying method, bag weight perception, sitting posture, electronic device usage posture, daily screen time, parental awareness of spinal health, living arrangements, and sports participation.

### Variable definitions

The primary outcome was post-exercise ATR≥7°, selected because: (a) the prevalence falls within the internationally reported range of 3%–10%; and (b) significant measurement clustering exists at ATR = 5°, suggesting that the ≥5° threshold is vulnerable to measurement bias. The secondary outcome was initial screening ATR≥5°.

Fifteen variables were coded for analysis: sex (female vs. male), age (continuous), BMI (continuous), school type (secondary vs. primary), family history (yes vs. no), bag carrying method (non-bilateral vs. bilateral), bag weight (heavy vs. normal), sitting posture (poor vs. good), electronic device posture (non-upright vs. upright), screen time (hours/day, continuous), parental awareness (low vs. high), living arrangement (non-parental vs. parental), sports participation (yes vs. no), menarche status (for females only), and menarche age (for females only).

### Statistical analysis

Descriptive statistics were calculated for all variables. Univariate logistic regression was performed for the primary outcome, with variables showing *P* < 0.20 considered as candidates for the multivariable model. The number of variables in the multivariable model was limited by the events per variable (EPV) criterion (EPV≥10), yielding a maximum of 5 variables for the primary outcome (50 events/10 = 5.0). Variable selection was optimized using Akaike's Information Criterion (AIC).

Model performance was evaluated using the area under the receiver operating characteristic curve (AUC), Hosmer-Lemeshow goodness-of-fit test, variance inflation factor (VIF) for multicollinearity, and decision curve analysis (DCA). Four sensitivity analyses were conducted: (1) main analysis (ATR≥7°); (2) ATR≥5°; (3) excluding students with ATR = 5°; and (4) post-exercise ATR≥5°. Subgroup analyses were performed stratified by school type, sex, and age group, with interaction terms tested.

The effectiveness of the spinal movement test was evaluated by calculating reclassification rates at each ATR threshold. To statistically validate the observed clustering at ATR = 5°, a chi-square goodness-of-fit test was performed, comparing the observed frequency at ATR = 5° against the expected frequency estimated by linear interpolation from adjacent ATR values. Statistical analyses were performed using R version 4.3.0 and Python version 3.11. A two-sided *P* < 0.05 was considered statistically significant.

### Sample size justification

With 50 events (post-exercise ATR≥7°) and 5 predictor variables, the EPV was 10, exceeding the recommended minimum of 10, providing adequate statistical power for the multivariable logistic regression model.

## Results

### Participant characteristics

A total of 813 students (408 males, 405 females) aged 10–17 years (mean 12.7 ± 1.8 years) participated in the study. There were 400 primary school students (49.2%) and 413 secondary school students (50.8%). The mean BMI was 19.9 ± 4.3 kg/m^2^. Table 1 presents the demographic and clinical characteristics stratified by screening outcome.

**Table 1:** Demographic and clinical characteristics by screening outcome.

Variable	Normal (*n* = 533)	Poor posture (*n* = 65)	Screen-positive (*n* = 215)	*P*
Age (years)	12.7 ± 1.8	12.7 ± 1.8	12.7 ± 1.7	0.977
BMI (kg/m^2^)	20.1 ± 4.3	19.6 ± 4.5	19.5 ± 4.3	0.162
Height (m)	1.56 ± 0.12	1.56 ± 0.12	1.57 ± 0.12	0.934
Weight (kg)	49.8 ± 13.7	48.4 ± 14.8	48.1 ± 13.8	0.272
Female sex	253 (47.5%)	49 (75.4%)	103 (47.9%)	<0.001
Secondary school	265 (49.7%)	36 (55.4%)	112 (52.1%)	0.625
Family history	17 (3.6%)	2 (3.6%)	6 (3.5%)	0.996
Non-bilateral bag	38 (7.2%)	4 (6.2%)	22 (10.2%)	0.327
Heavy bag	278 (54.6%)	42 (66.7%)	114 (53.8%)	0.166
Poor sitting posture	319 (62.1%)	42 (67.7%)	141 (66.5%)	0.415
Non-upright screen	283 (55.1%)	35 (55.6%)	123 (58.6%)	0.686
Low parental awareness	210 (41.2%)	23 (35.9%)	82 (38.3%)	0.870

BMI, body mass index. Continuous variables are presented as mean ± standard deviation; categorical variables as *n* (%).

### Screening results

Of the 813 participants, 533 (65.6%) were classified as normal, 65 (8.0%) as poor posture, and 215 (26.4%) as screen-positive. Among the 215 screen-positive students, 165 (76.7%) were Grade I, 40 (18.6%) were Grade II, and 10 (4.7%) were Grade III. Table 2 presents the distribution of ATR values.

**Table 2:** Distribution of angle of trunk rotation (ATR) measurements.

ATR (°)	*n* (%)	ATR (°)	*n* (%)	Summary
0	569 (70.0%)	9	7 (0.9%)	≥5°: 218 (26.8%)
1	1 (0.1%)	10	4 (0.5%)	≥7°: 50 (6.1%)
2	3 (0.4%)	11	5 (0.6%)	≥10°: 12 (1.5%)
3	2 (0.2%)	12	1 (0.1%)	
4	20 (2.5%)	14	1 (0.1%)	
5	144 (17.7%)	17	1 (0.1%)	
6	19 (2.3%)			
7	25 (3.1%)			
8	11 (1.4%)			

ATR, angle of trunk rotation.

### Statistical validation of the clustering at ATR = 5°

To determine whether the observed clustering at ATR = 5° exceeded what would be expected under a smooth distribution, a chi-square goodness-of-fit test was performed. Under the null hypothesis that ATR measurements follow a monotonically decreasing distribution without preferential accumulation at any specific value, the expected frequency at ATR = 5° was estimated by linear interpolation from the adjacent values at ATR = 4° and ATR = 6°. The observed frequency at ATR = 5° (*n* = 144, 17.7% of all participants) was dramatically higher than the interpolated expectation (*n* = 19.5), yielding a highly significant chi-square statistic (*χ*^2^ = 794.88, df = 1, *P* < 0.001). The ratio of ATR = 5° to the average per-degree frequency across ATR 1°–4° and 6°–9° was 13.1:1 (95% CI: 11.1–15.4), confirming substantial and statistically significant aggregation at this threshold. The distribution of ATR values and the pre/post comparison are shown in Figure 1.

**Figure 1:**
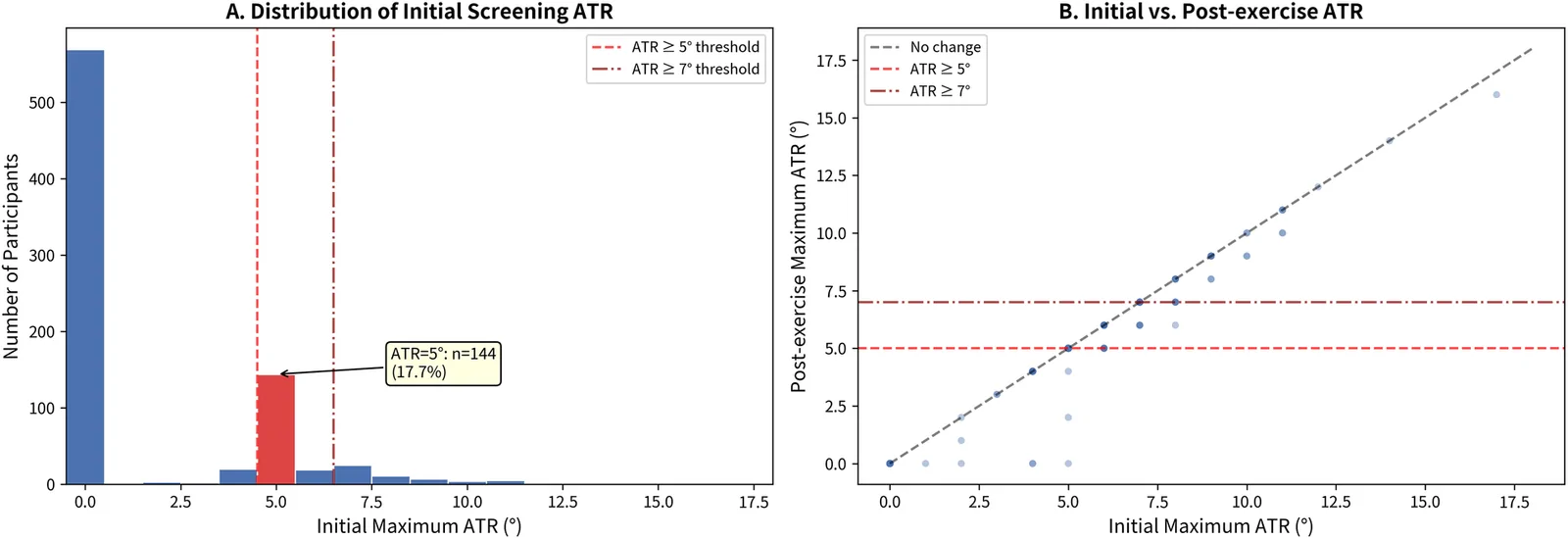
Distribution of angle of trunk rotation (ATR) among 813 students. **(A)** Distribution of initial screening ATR; a significant clustering is observed at ATR = 5° (144 students, 17.7%). **(B)** Initial versus post-exercise maximum ATR.

### Sex differences

Significant sex differences were observed across ATR thresholds. For ATR≥5°, the prevalence was 27.7% in males vs. 25.9% in females (*P* = 0.570). For ATR≥7°, the prevalence was 2.9% in males vs. 10.6% in females (*P* < 0.001). For ATR≥10°, the prevalence was 0.5% in males vs. 2.5% in females (*P* = 0.028). The distribution of grades and the grade-by-sex comparison are shown in Figure 2.

**Figure 2:**
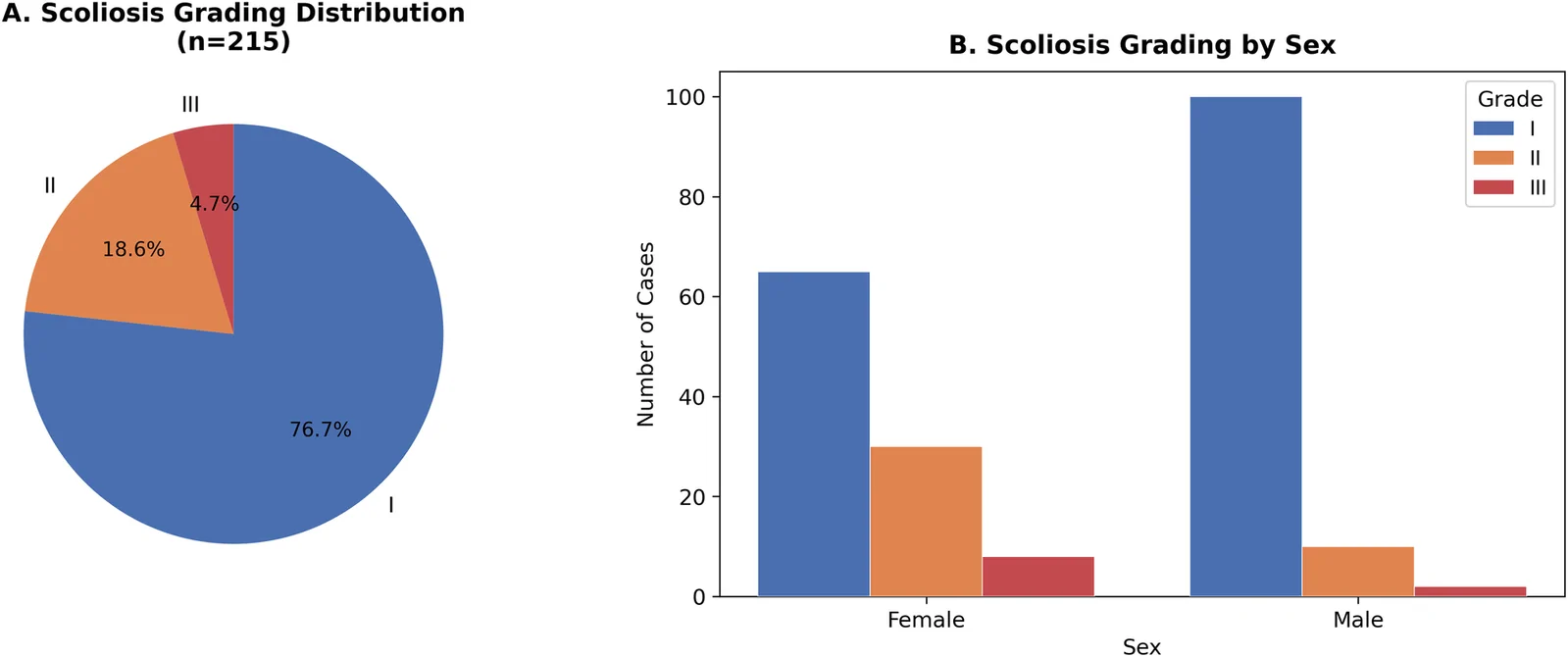
Scoliosis grading by sex. **(A)** Scoliosis grading distribution (*n* = 215). **(B)** Scoliosis grading by sex.

### Univariate analysis

Table 3 presents the results of univariate logistic regression for the primary outcome (ATR≥7°). Variables with *P* < 0.20 and included in the multivariable model were: female sex (OR = 3.575, *P* < 0.001), BMI (OR = 0.925, *P* = 0.039), non-bilateral bag use (OR = 1.612, *P* = 0.197), non-upright screen posture (OR = 1.473, *P* = 0.190), and screen time (OR = 1.422, *P* = 0.008).

**Table 3:** Univariate logistic regression for ATR≥7° (*n* = 813).

Variable	*β*	SE	OR (95% CI)	P
Female sex	1.274	0.288	3.575 (2.032–6.288)	<0.001
Age (years)	0.075	0.103	1.078 (0.881–1.318)	0.467
BMI (kg/m^2^)	-0.078	0.038	0.925 (0.859–0.996)	0.039
Secondary school	0.104	0.297	1.110 (0.621–1.984)	0.726
Family history	0.083	0.602	1.087 (0.334–3.539)	0.891
Non-bilateral bag	0.477	0.370	1.612 (0.779–3.332)	0.197
Heavy bag	0.037	0.299	1.037 (0.578–1.862)	0.902
Poor sitting posture	0.191	0.318	1.210 (0.649–2.256)	0.548
Non-upright screen	0.387	0.295	1.473 (0.826–2.627)	0.190
Screen time (h/day)	0.352	0.132	1.422 (1.098–1.841)	0.008

SE, standard error; OR, odds ratio; CI, confidence interval; BMI, body mass index.

### Multivariable logistic regression

The main model (Table 4) included 5 variables selected by AIC optimization from 776 participants with complete data. Female sex (OR = 3.509, 95%CI: 1.917–6.425, *P* < 0.001) and screen time (OR = 1.391, 95%CI: 1.070–1.809, *P* = 0.013) remained significantly associated with ATR≥7°. BMI showed marginal significance (OR = 0.930, 95%CI: 0.865–1.001, *P* = 0.052). Non-upright screen posture (OR = 1.403, *P* = 0.260) and non-parental living (OR = 0.377, *P* = 0.109) were included based on clinical relevance. The model showed acceptable fit (AIC = 358.9, Pseudo R^2^ = 0.078, LLR *P* < 0.001). Adjusted odds ratios for the main model are presented in Figure 3.

**Table 4:** Multivariable logistic regression - main model (ATR≥7°, *n* = 776).

Variable	β	OR (95% CI)	P
Female sex	1.255	3.509 (1.917–6.425)	<0.001
BMI (kg/m^2^)	−0.073	0.930 (0.865–1.001)	0.052
Non-upright screen	0.338	1.403 (0.777–2.535)	0.260
Screen time (h/day)	0.330	1.391 (1.070–1.809)	0.013
Non-parental living	−0.976	0.377 (0.114–1.243)	0.109

Model statistics: AIC = 358.9, Pseudo R^2^ = 0.078, LLR *P* < 0.001. SE, standard error; OR, odds ratio; CI, confidence interval; BMI, body mass index.

**Figure 3:**
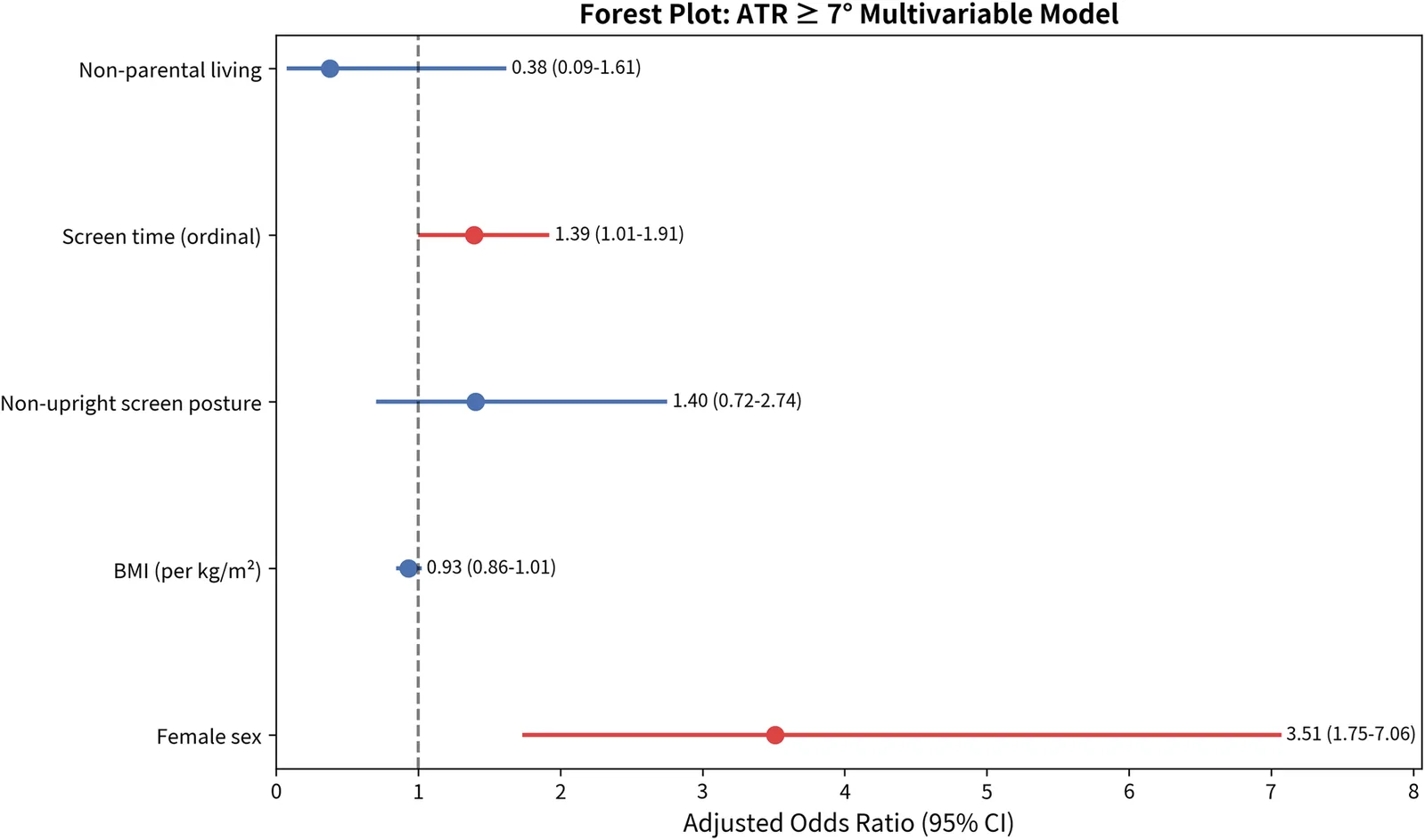
Forest plot of odds ratios for the main model.

The secondary model using ATR≥5° as the outcome (Table 5) included 13 variables from 658 participants. No variable reached statistical significance, and the model showed poor fit (Pseudo R^2^ = 0.014, LLR *P* = 0.638), indicating that the 13 selected variables had limited ability to explain variation in screen-positive status at the ATR≥5° threshold. This does not imply that the ATR≥5° threshold itself lacks screening utility; rather, it suggests that the questionnaire-based variables included in this study are insufficient to characterize the factors associated with ATR≥5° positivity.

**Table 5:** Multivariable logistic regression - full model (ATR≥5°, *n* = 658).

Variable	β	OR (95% CI)	P
Female sex	0.124	1.132 (0.787–1.628)	0.503
Age (years)	0.032	1.032 (0.921–1.157)	0.589
BMI (kg/m^2^)	−0.012	0.988 (0.950–1.028)	0.560
Secondary school	−0.062	0.940 (0.641–1.378)	0.744
Family history	0.201	1.223 (0.514–2.908)	0.646
Non-bilateral bag	0.365	1.441 (0.831–2.498)	0.193
Heavy bag	−0.029	0.971 (0.671–1.406)	0.881
Poor sitting posture	0.185	1.203 (0.829–1.747)	0.330
Non-upright screen	0.148	1.159 (0.803–1.673)	0.432
Screen time (h/day)	0.048	1.049 (0.920–1.196)	0.481
Low parental awareness	−0.113	0.893 (0.622–1.283)	0.541
Non-parental living	−0.312	0.732 (0.383–1.399)	0.347
Sports participation	0.112	1.119 (0.738–1.696)	0.587

Model statistics: Pseudo R^2^ = 0.014, LLR *P* = 0.638.

### Model performance

Table 6 compares the discrimination and calibration of the main model versus the full model. The main model demonstrated acceptable discrimination (AUC = 0.714 vs. 0.584) and good calibration (Hosmer-Lemeshow *P* = 0.680 vs. 0.250). Maximum VIF was 4.01, indicating no significant multicollinearity. ROC curves are shown in Figure 4, calibration plots in Figure 5, and decision curve analysis in Figure 6.

**Table 6:** Model performance comparison.

Metric	Main Model (ATR≥7°)	Full Model (ATR≥5°)
AUC	0.714	0.584
Pseudo R^2^	0.078	0.014
Hosmer-Lemeshow P	0.680	0.250
Maximum VIF	4.01	3.87
Events per variable	10	12.5

AUC, area under the receiver operating characteristic curve; VIF, variance inflation factor.

**Figure 4:**
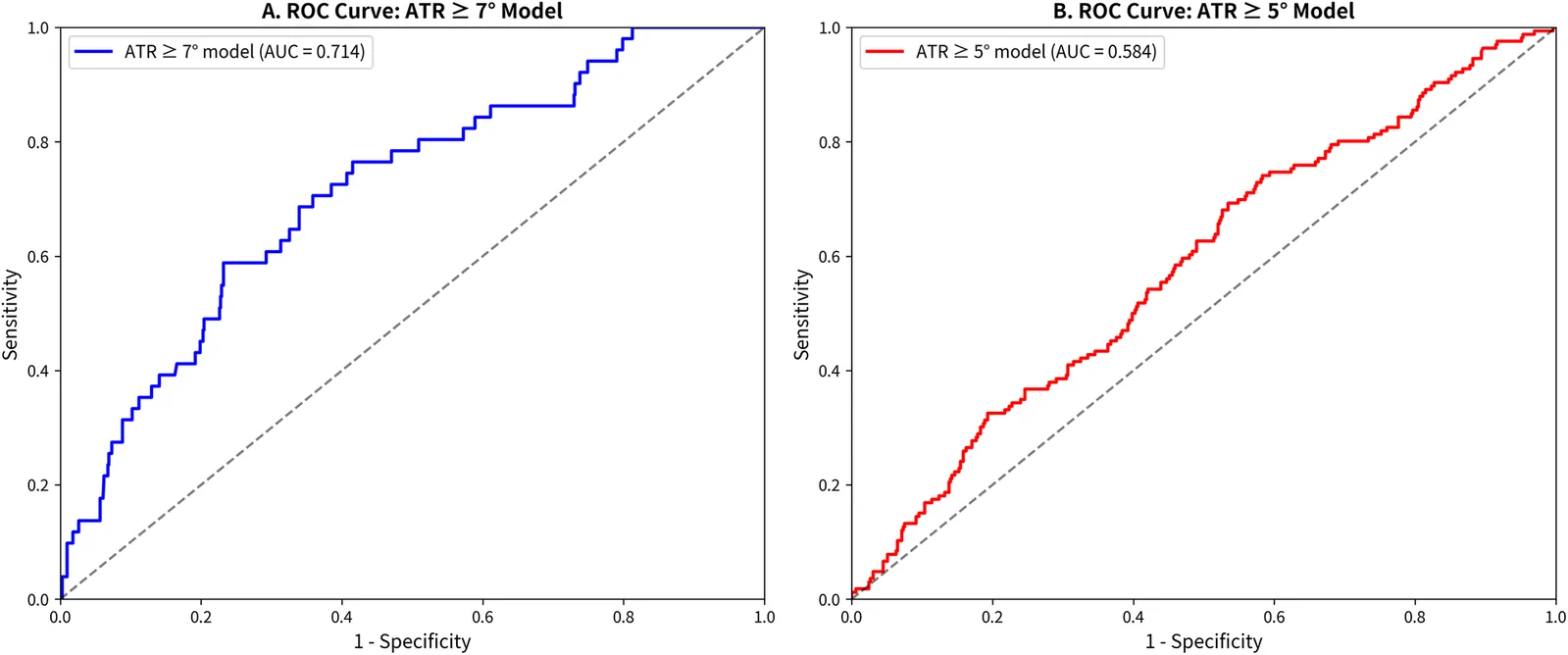
ROC curves for main and full models. **(A)** ATR ≥ 7° model. **(B)** ATR ≥ 5° model.

**Figure 5:**
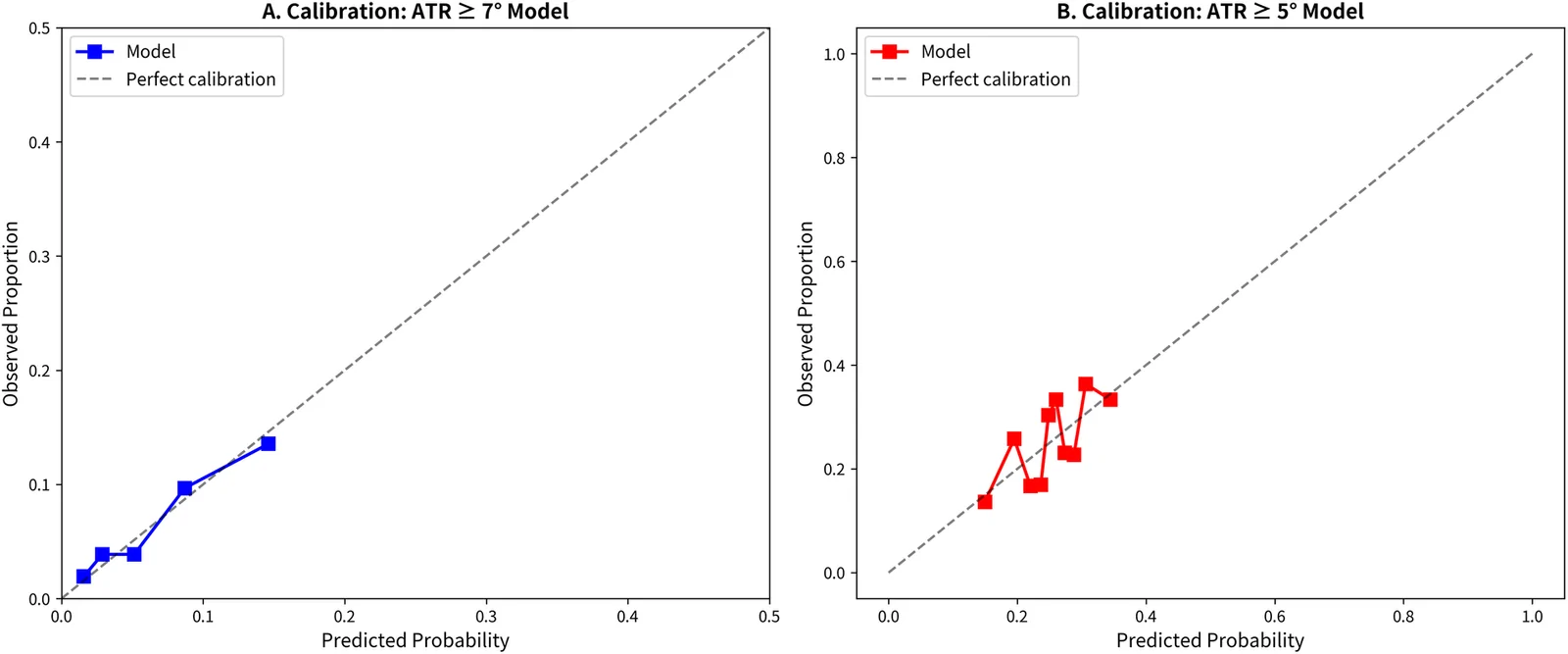
Calibration plots. **(A)** ATR ≥ 7° model. **(B)** ATR ≥ 5° model.

**Figure 6:**
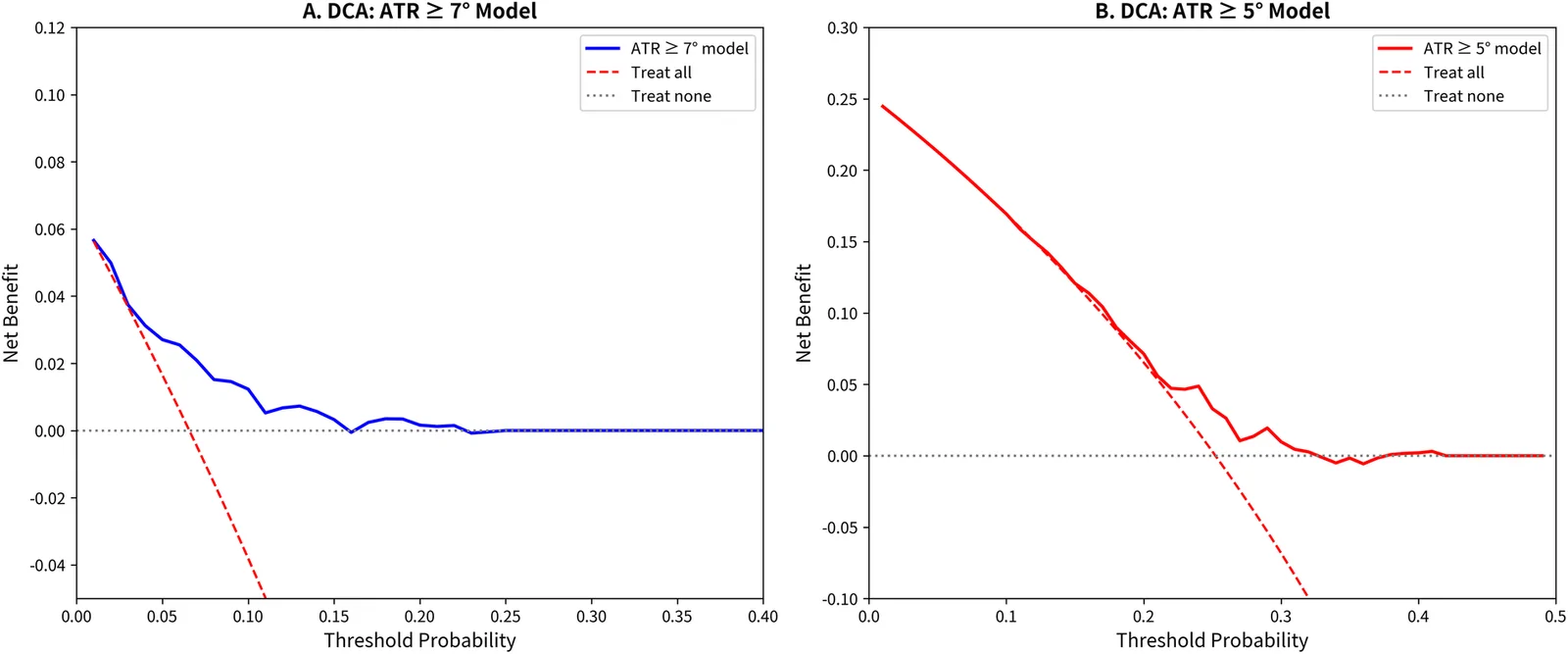
Decision curve analysis. **(A)** ATR ≥ 7° model. **(B)** ATR ≥ 5° model.

### Spinal movement test

Of 813 participants, 280 (34.4%) with initial ATR≥5° underwent the spinal movement test. Table 7 presents the reclassification results. The reclassification rates were low: 1.4% for ATR≥5°, 9.1% for ATR≥7°, and 16.7% for ATR≥10°. The mean ATR change after exercise was −0.16°, with 31 students (11.1%) showing ATR reduction, 249 (89.0%) showing no change, and no students showing ATR increase. The effects of the spinal movement test on ATR are shown in Figure 7.

**Table 7:** Spinal movement test results (*n* = 280).

Threshold	Pre-exercise (n)	Reclassified (n, %)	Mean ATR Change (°)
≥5°	218	3 (1.4%)	−0.16
≥7°	55	5 (9.1%)	−0.21
≥10°	12	2 (16.7%)	−0.33

ATR, angle of trunk rotation.

**Figure 7:**
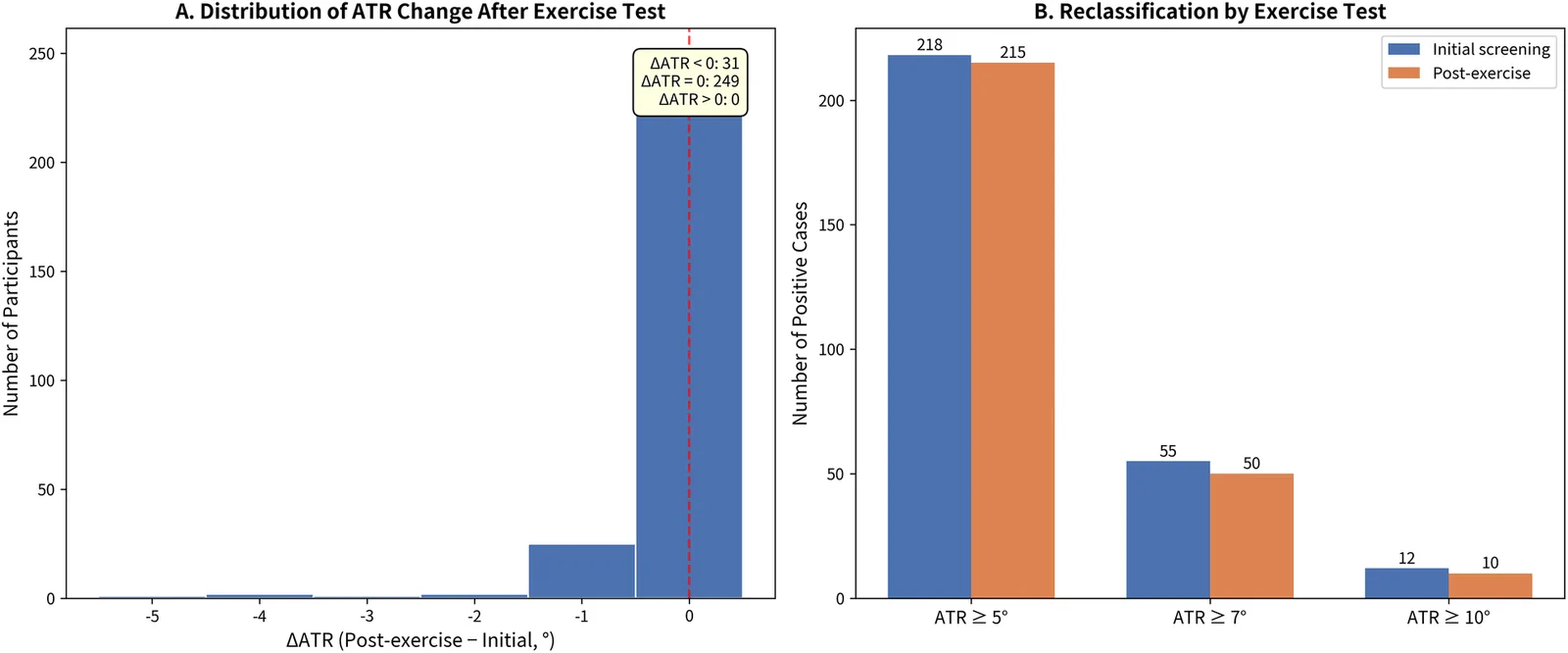
Effects of the spinal movement test on ATR measurements. **(A)** Distribution of ATR change after the exercise test. **(B)** Reclassification by the exercise test.

### Sensitivity analyses

Table 8 presents the results of four sensitivity analyses. The main analysis and analysis excluding ATR = 5° identified the same significantly associated factors (female sex and screen time), confirming the robustness of these findings. The analyses using ATR≥5° and post-exercise ATR≥5° identified no significant variables, consistent with the poor model fit observed in the full model.

**Table 8:** Sensitivity analyses.

Analysis	n	Cases	Significant Variables
Main analysis (ATR≥7°)	776	50	Female, Screen time
ATR≥5°	658	163	None
Excluding ATR = 5°	632	55	Female, Screen time
Post-exercise ATR ≥ 5°	649	160	None

ATR, angle of trunk rotation.

### Subgroup analyses

Table 9 presents the results of subgroup analyses. Female sex was consistently associated with screen-positive status across most subgroups. Screen time showed significant association only in females (OR = 1.70, *P* = 0.006), with a significant sex × screen time interaction (OR = 1.68, *P* = 0.046). BMI showed significant association only in secondary school students (OR = 0.90, *P* = 0.029).

**Table 9:** Subgroup analyses.

Subgroup	Variable	OR (95% CI)	*P*	*P* interaction
Primary school	Female sex	3.62 (1.21–10.82)	0.021	0.952
Primary school	Screen time	1.65 (1.06–2.57)	0.027	
Secondary school	Female sex	3.49 (1.64–7.43)	0.001	
Secondary school	BMI	0.90 (0.82–0.99)	0.029	
Males	BMI	0.89 (0.79–1.00)	0.052	0.049
Males	Screen time	1.03 (0.77–1.37)	0.849	
Females	BMI	0.96 (0.88–1.05)	0.369	
Females	Screen time	1.70 (1.17–2.48)	0.006	
Age 10–12	Female sex	2.89 (1.03–8.11)	0.043	0.781
Age 10–12	Non-upright screen	2.01 (0.89–4.54)	0.094	
Age 13–15	Female sex	4.12 (1.89–8.97)	<0.001	
Age 13–15	BMI	0.91 (0.83–0.99)	0.036	
Age 16–17	Female sex	2.98 (0.62–14.31)	0.171	0.612
Age 16–17	Heavy bag	2.45 (0.87–6.91)	0.092	
Overall	Female × Screen time	1.68 (1.01–2.79)	0.046	0.049

OR, odds ratio; CI, confidence interval; BMI, body mass index.

## Discussion

### The apparent 5° measurement clustering

This study identified a statistically significant measurement clustering at ATR = 5°, with 17.7% of all participants having this exact measurement. This represents a 5.5-fold increase compared to the combined ATR 1–4° category, and chi-square goodness-of-fit testing showed that this aggregation far exceeded what would be expected under a smooth distribution (*χ*^2^ = 794.88, *P* < 0.001). The ratio of ATR = 5° to ATR = 6° was 7.6:1, which is far beyond what would be expected from a continuous measurement distribution. This apparent clustering phenomenon has important implications for scoliosis screening programs.

Several mechanisms may plausibly contribute to the observed aggregation. Manual angle measurements in orthopedic practice are commonly recorded in 5° increments (23), and examiners may tend to round values near the psychologically salient 5° cutoff to that value; additionally, students may instinctively adjust their posture when being measured near the threshold. To our knowledge, no previous study has reported terminal digit preference or 5° clustering in ATR measurements; the earlier scoliometer studies (24, 25) addressed measurement validity and reliability, not digit preference. The present study is therefore the first to formally quantify the magnitude of this aggregation, although the present data cannot determine its cause. It should be noted that the chi-square analysis used here relies on linear interpolation to estimate expected frequencies, which may not capture the true underlying distribution of ATR measurements. Accordingly, this analysis serves as a descriptive quantification of the observed aggregation rather than a confirmatory test of its origin. The clustering may reflect a combination of examiner rounding to a psychologically salient threshold and natural postural adjustments near commonly used cutoffs; controlled studies with blinded or instrument-based measurements would be needed to disentangle these explanations.

Using ATR≥5°, the prevalence was 26.8%, substantially higher than international estimates of 0.47%–5.2% (2). In contrast, using ATR≥7°, the prevalence was 6.8%, which falls within the expected range. This marked difference underscores the importance of selecting appropriate thresholds and suggests that the apparent 5° clustering may contribute to overestimation of scoliosis prevalence in some screening programs.

### Prevalence and threshold dependence

Our findings demonstrate that prevalence estimates are highly dependent on the chosen ATR threshold. The 4.4-fold difference in prevalence between ATR≥5° (26.8%) and ATR≥7° (6.8%) highlights the need for standardized screening criteria. International guidelines vary considerably: the American Orthopaedic Society recommends ≥5°, while SOSORT recommends ≥7° (10, 13). The Chinese national guidelines suggest using ≥5° for initial screening with clinical correlation required for diagnosis (12).

A recent population-wide analysis of 2.22 million adolescents in mainland China reported a definitive scoliosis prevalence of 1.20% (Cobb angle ≥10° confirmed by radiography), with a primary screening positive rate of 3.97% (4). Another meta-analysis of 42 studies involving over 1.1 million subjects reported an overall prevalence of 1.2% among Chinese adolescents (5). Our finding of 6.8% using ATR≥7° is higher than these estimates because ATR-based screening identifies both structural and non-structural curves, and not all screen-positive cases are confirmed by radiography.

### Associated factors

Female sex showed the strongest association with screen-positive status (OR = 3.509, *P* < 0.001), consistent with previous studies (2, 3). The sex difference was particularly pronounced at higher ATR thresholds: 2.9% vs. 10.6% for ATR≥7° and 0.5% vs. 2.5% for ATR≥10°.

Screen time was significantly associated with screen-positive status (OR = 1.391, *P* = 0.013), consistent with recent studies (20, 21). Importantly, we found a significant sex × screen time interaction (OR = 1.68, *P* = 0.046), with screen time showing a stronger effect in females (OR = 1.70, *P* = 0.006) compared to males (OR = 1.03, *P* = 0.849). This suggests that females may be more vulnerable to the adverse effects of prolonged screen use, possibly due to differences in posture, muscle strength, or hormonal factors.

BMI showed marginal significance as a potential protective factor (OR = 0.930, *P* = 0.052), consistent with studies reporting lower BMI in AIS patients (16–18). However, this relationship may reflect detection bias rather than a true protective effect of higher BMI. BMI affects scoliometer readings, with higher BMI yielding lower ATR measurements, potentially masking underlying asymmetries (19). The non-significance of BMI in most subgroup analyses supports this interpretation.

Other factors including family history, bag carrying habits, sitting posture, and parental awareness showed no significant associations, which may be due to limited sample size or recall bias in self-reported measures.

### Spinal movement test effectiveness

The spinal movement test showed limited effectiveness in reducing false positives. Only 9.1% of students with ATR≥7° were reclassified after exercise, with a mean ATR change of only −0.21°. This suggests that most students with positive screening results have genuine trunk rotation rather than postural artifacts. The test may be more useful at the ATR≥10° threshold, where 16.7% were reclassified.

### Clinical implications

Based on our findings, we propose the following recommendations: (1) Use ATR≥7° as the referral threshold rather than ≥5° to reduce the impact of measurement clustering and unnecessary referrals. (2) Implement training programs to reduce measurement clustering, including instructions to avoid rounding to 5° increments. (3) Give special attention to female students, particularly during puberty, and emphasize screen time counseling. (4) Use clinical correlation rather than single ATR measurements for decision-making.

### Limitations

Several limitations should be acknowledged. First, this was a cross-sectional study, which cannot establish causality; accordingly, we use the term “associated factors” rather than “risk factors” throughout the manuscript. Second, radiographs were not obtained to confirm the diagnosis with Cobb angle measurements, and we cannot therefore determine the diagnostic accuracy of different ATR thresholds in this population. Third, the study was conducted in a single city, which may limit generalizability. Fourth, lifestyle factors were based on self-report, subject to recall bias. Fifth, the sample size was limited for subgroup analyses. Sixth, formal inter-rater and intra-rater reliability metrics were not calculated. Although each participant was primarily examined by a trained rehabilitation physician with supervisory oversight by an attending physician, the absence of formal reliability testing is a limitation that may affect the precision of ATR measurements. The original dataset retained only the final measurement value, precluding *post-hoc* reliability analysis. Future screening studies should incorporate standardized reliability protocols, including pre-study inter-rater calibration and duplicate measurements on a subset of participants, to strengthen the methodological rigor of scoliosis screening research.

## Conclusion

This study identifies statistically significant measurement clustering at the 5° ATR threshold (*χ*^2^ = 794.88, *P* < 0.001), which substantially inflates prevalence estimates when using the ≥5° threshold. Using ATR≥7° yields a screen-positive prevalence (6.8%) that is more consistent with international data derived from radiographic confirmation studies. However, as this study did not include radiographic verification, the relative diagnostic accuracy of different ATR thresholds cannot be determined. Female sex and screen time were independently associated with screen-positive status, with a significant sex × screen time interaction suggesting that females may be more vulnerable to the adverse effects of prolonged screen use. These findings have important implications for the design and interpretation of scoliosis screening programs.

## Data Availability

The raw data supporting the conclusions of this article will be made available by the authors, without undue reservation.

## References

[1] Cheng JC Castelein RM Chu WC Danielsson AJ Dobbs MB Grivas TB. Adolescent idiopathic scoliosis. Nat Rev Dis Primers. (2015) 1:15063. 10.1038/nrdp.2015.63 27188385

[2] Konieczny MR Senyurt H Krauspe R. Epidemiology of adolescent idiopathic scoliosis. J Child Orthop. (2013) 7(1):3–9. 10.1007/s11832-012-0457-4 24432052 PMC3566258

[3] Weinstein SL Dolan LA Cheng JC Danielsson A Morcuende JA. Adolescent idiopathic scoliosis. Lancet. (2008) 371(9623):1527–37. 10.1016/S0140-6736(08)60658-3 18456103

[4] Xu S Li K Jin L Dong Y Liang Y Liu C. Distribution of scoliosis in 2.22 million adolescents in mainland China: a population-wide analysis. J Glob Health. (2024) 14:04117. 10.7189/jogh.14.04117 39026457 PMC11258535

[5] Fu X Meng S Huang X Li W Ye B Chen S. The prevalence of scoliosis among adolescents in China: a systematic review and meta-analysis. J Orthop Surg Res. (2024) 19(1):585. 10.1186/s13018-024-05077-0 39342221 PMC11437733

[6] Negrini S Donzelli S Aulisa AG Czaprowski D Schreiber S De Mauroy JC. 2016 SOSORT guidelines: orthopaedic and rehabilitation treatment of idiopathic scoliosis during growth. Scoliosis Spinal Disord. (2018) 13:3. 10.1186/s13013-017-0145-8 29435499 PMC5795289

[7] Luk KDK Lee CF Cheung KMC Cheng JCY Ng BKW Lam TP. Clinical effectiveness of school screening for adolescent idiopathic scoliosis: a large population-based retrospective cohort study. Spine. (2010) 35(17):1607–14. 10.1097/BRS.0b013e3181c7cb8c 20453727

[8] Côté P Kreitz BG Cassidy JD Dzus AK Martel J. A study of the diagnostic accuracy and reliability of the scoliometer and adam’s forward bend test. Spine. (1998) 23(7):796–802. 10.1097/00007632-199804010-00011 9563110

[9] Murrell GA Coonrad RW Moorman CT 3rd Fitch RD. An assessment of the reliability of the scoliometer. Spine. (1993) 18(6):709–12. 10.1097/00007632-199305000-00006 8516699

[10] Bunnell WP. Selective screening for scoliosis. Clin Orthop Relat Res. (2005) 434:40–5. 10.1097/01.blo.0000163242.92733.66 15864030

[11] Karachalios T Sofianos J Roidis N Sapkas G Korres D Nikolopoulos K. Ten-year follow-up evaluation of a school screening program for scoliosis: is the forward-bending test an accurate diagnostic criterion for the screening of scoliosis? Spine. (1999) 24(22):2318–24. 10.1097/00007632-199911150-00006 10586455

[12] Chinese Orthopedic Association, Zhao Y Zhao Y Lu S Yang Q Chen L Ma X. Guideline for adolescent scoliosis screening in China (public version 2024). J Orthop Translat. (2025) 50:364–72. 40171107 10.1016/j.jot.2024.09.006 PMC11960534

[13] Negrini S Aulisa L Ferraro C Fraschini P Masiero S Simonazzi P. Italian guidelines on rehabilitation treatment of adolescents with scoliosis or other spinal deformities. Eura Medicophys. (2005) 41(2):183–201. 16200035

[14] Kleinig O To M-S Ovenden CD Kovoor JG Goh R Lam L. Vital sign measurements demonstrate terminal digit bias and boundary effects. Emerg Med Australas. (2024) 36:543–6. 10.1111/1742-6723.14395 38413380

[15] Tsuruda KM Hofvind S Akslen LA Hoff SR Veierød MB. Terminal digit preference: a source of measurement error in breast cancer diameter reporting. Acta Oncol. (2020) 59:260–7. 10.1080/0284186X.2019.1669817 31566467

[16] Burwell RG Clark EM Dangerfield PH Moulton A. Adolescent idiopathic scoliosis (AIS): a multifactorial cascade concept for pathogenesis and embryonic origin. Scoliosis Spinal Disord. (2016) 11:8. 10.1186/s13013-016-0063-1 27252984 PMC4888516

[17] Hershkovich O Friedlander A Gordon B Arzi H Derazne E Tzur D. Association between body mass index, body height, and the prevalence of spinal deformities. Spine J. (2014) 14(8):1581–7. 10.1016/j.spinee.2013.09.034 24332597

[18] Qiu Y Sun X Qiu X Li W Zhu Z Zhu F. Decreased circulating leptin level and its association with body and bone mass in girls with adolescent idiopathic scoliosis. Spine. (2007) 32(24):2703–10. 10.1097/BRS.0b013e31815a59e5 18007248

[19] Margalit A McKean G Constantine A Thompson CB Lee RJ Sponseller PD. Body mass hides the curve: thoracic scoliometer readings vary by body mass index value. J Pediatr Orthop. (2017) 37(4):e255–60. 10.1097/BPO.0000000000000899 27861214 PMC5422115

[20] Zhu L Ru S Wang W Dou Q Li Y Guo L. Associations of physical activity and screen time with adolescent idiopathic scoliosis. Environ Health Prev Med. (2023) 28:55. 10.1265/ehpm.23-00004 37766541 PMC10569969

[21] Hu M Zhang Z Zhou X Gao R Wang C Ma J. Prevalence and determinants of adolescent idiopathic scoliosis from school screening in huangpu district, Shanghai, China. Am J Transl Res. (2022) 14(6):4132–8. 35836864 PMC9274610

[22] Akçay B Çolak TK Apti A Çolak İ. The immediate effect of hanging exercise and muscle cylinder exercise on the angle of trunk rotation in adolescent idiopathic scoliosis. Healthcare. (2024) 12(3):305. 10.3390/healthcare12030305 PMC10855408 38338189

[23] Kage T Inui H Tomita T Yamazaki T Taketomi S Yamagami R. Weight-bearing knee flexion angle better correlates with patient-reported outcome measures than non-weight-bearing condition in total knee arthroplasty: a three-dimensional analysis study. BMC Musculoskelet Disord. (2021) 22:718. 10.1186/s12891-021-04594-x 34419014 PMC8380316

[24] Amendt LE Ause-Ellias KL Eybers JL Wadsworth CT Nielsen DH Weinstein SL. Validity and reliability testing of the scoliometer. Phys Ther. (1990) 70(2):108–17. 10.1093/ptj/70.2.108 2296610

[25] Bonagamba GH Coelho DM Oliveira AS Inter and intra-rater reliability of the scoliometer. Rev Bras Fisioter. 2010;14(5):432–8. 10.1590/S1413-35552010005000025 21049239

